# Early Mobilization in the Pediatric Intensive Care Unit: A Quality Improvement Initiative

**DOI:** 10.1097/pq9.0000000000000256

**Published:** 2020-01-31

**Authors:** Jodi M. Herbsman, Michael D’Agati, Daniella Klein, Siobhan O’Donnell, John R. Corcoran, Tiffany D. Folks, Yasir M. Al-Qaqaa

**Affiliations:** From the * Rusk Rehabilitation, NYU Langone Health, New York, N.Y.; † Department of Nursing, NYU Langone Health, New York, N.Y.; ‡ Department of Pediatrics, NYU Langone Health, New York, N.Y.

## Abstract

Mobilizing patients during an intensive care unit admission results in improved clinical and functional outcomes. The goal of this quality improvement project was to increase the percentage of patients in the pediatric intensive care unit (PICU) mobilized early from 62% to 80%. Early mobilization was within 18 hours of admission for nonmechanically ventilated (non-MV) patients and 48 hours for mechanically ventilated (MV) patients.

**Methods:**

We collected data from September 15, 2015, to December 15, 2016, identified key drivers and barriers, and developed interventions. Interventions included the development of an algorithm to identify patients appropriate for mobilization, management of barriers to mobilization, and education on the benefits of early mobilization. The percentage of PICU patients mobilized early; the percentage of patients with physical therapy, occupational therapy (OT), speech-language pathology (SLP), and activity orders; identified barriers; PICU and hospital length of stay (LOS) and discharge disposition, were compared between the pre- and postintervention groups and the non-MV and MV subgroups. The MV subgroup was too small for statistical testing.

**Results:**

All measures in the combined postintervention group improved and reached significance (<0.05), except for the percentage of SLP orders and discharged home. Percentage mobilized early increased 25%, activity orders 50%, physical therapist orders 14%, OT orders 11%, SLP orders 7%, and discharged home 6%. Hospital LOS decreased by 35%, and PICU LOS decreased by 34%. All measures in the postintervention, non-MV subgroup improved and reached significance (<0.05).

**Conclusions:**

This early mobilization program was associated with statistically significant improvements in the rate of early mobilization, activity and therapy orders, and hospital and PICU LOS.

## INTRODUCTION

Patients in an intensive care unit (ICU) who are immobilized for, mechanically ventilated (MV), and/or sedated for a prolonged period experience physical, cognitive, and functional impairments and decreased quality of life.^1–8^ These deleterious effects can lead to associated conditions such as postintensive care syndrome (1), iatrogenic immobilization injuries, ICU-induced myopathy and neuropathy, and ICU-acquired weakness (2−5). Initial studies in children have demonstrated that pediatric patients who have survived critical illness are at risk for physical, cognitive, and functional impairments and decreased quality of life long after discharge from the pediatric intensive care unit (PICU; 5−8). Children who survive critical illness may have difficulty in school and social settings due to neurocognitive deficits and/or psychological illness.^5,7^ Early mobilization for adult ICU patients is safe and effective in preventing many of these adverse outcomes.^1,9–12^ Recent studies in the pediatric population have supported the safety, feasibility, and potential positive effects of early mobilization.^5,13,14^

Barriers to mobilization in the ICU have been documented.^5,14–17^ Based on adult literature, we performed a gap analysis to determine if patients in the PICU were being mobilized timely. The gap analysis highlighted the limited mobilization of critically ill children. Barriers to mobilizing critically ill children include lack of physician orders, inadequate equipment, lack of guidelines for mobilization and sedation, medical complexity of the child, insufficient resources, and fear of endotracheal tube dislodgement.^14,15^

We implemented an early mobilization program in the PICU by harnessing an interdisciplinary and patient/family-centered team. Our primary aim was to improve the rate of early mobilization to 80%. We sought to improve several process measures, including the percentage of patients with physical therapist (PT), occupational therapist (OT), speech-language pathologist (SLP), and activity orders, and removing barriers to mobilization with secondary aims to decrease length of stay (LOS) and increase the percentage of patients discharged home.

## METHODS

### Context

Hassenfeld Children’s Hospital is a 110-bed pediatric acute care hospital within NYU Langone Health, a 705-bed academic medical center. The PICU is a 12-bed unit that treats a variety of medical diagnoses including, but not limited to, postoperative orthopedic and neurosurgical patients, medically complex patients, and patients in respiratory distress. The hospital is located in an urban environment with affiliated inpatient and outpatient rehabilitation.

Patients 18 months and older admitted to the PICU from December 16, 2015, to December 15, 2016, received the various interventions. We excluded patients from data collection if they were too critically ill to be mobilized throughout their PICU stay, or if any component of the data was not available. Data were collected weekly on the percent of patients mobilized. Pre- and postintervention data were collected to determine if increasing rates of mobilization could be correlated with decreased LOS and increased percentage of patients discharged home.

The local institutional review board reviewed this study and deemed it to be a quality improvement project and not human subjects research.

### Interventions

#### Planning the Intervention

In July 2015, we met with potential stakeholders to ensure leadership support. We performed a gap analysis to determine if patients were being mobilized timely. We collected baseline data from September 15, 2015, to December 15, 2015, that included the percentage of patients with PT, OT, SLP and activity orders, time to the first mobilization, PICU and hospital LOS, discharge disposition, and date and time of PICU admission. Activity orders inform the team as to the type of mobilization the patient is cleared for, ranging from bed rest to ambulation. Baseline data showed that 62% of patients were being mobilized within the established time frames of 18 hours for non-MV patients and 48 hours for MV patients.

We formed an interdisciplinary group, consisting of physicians, residents, nurses, patient care technicians (PCTs), PTs, OTs, SLPs, respiratory therapists (RT), child life therapists, and senior family advisors (SFAs). SFAs are a trained group of employees whose children were previously treated at Hassenfeld Children’s Hospital. SFAs provide an indispensable perspective of the patients’ experience.

**Fig. 1.**
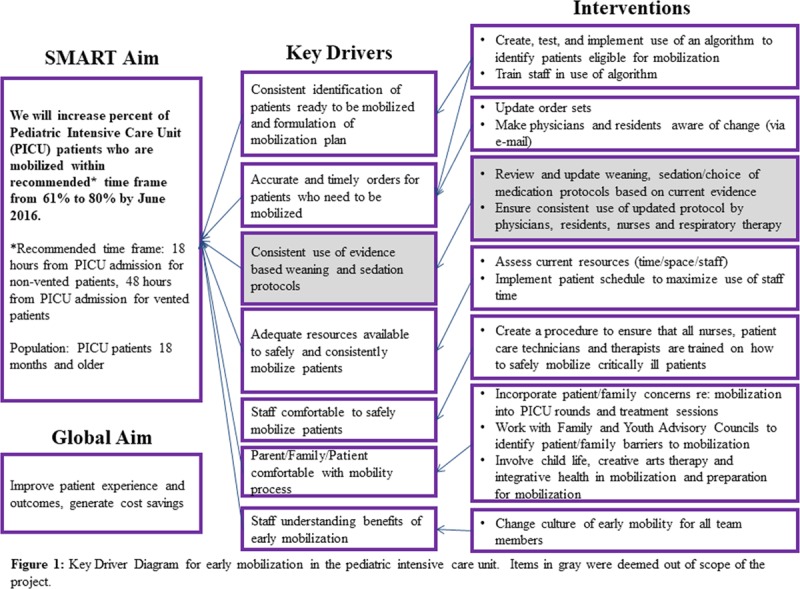
Key driver diagram for early mobilization in the pediatric intensive care unit. Items in gray were deemed out of scope of the project.

The team agreed on the aims of the project, as well as key drivers and interventions (Fig. 1). We identified the following key drivers: method to identify patients ready to be mobilized, accurate and timely orders, adequate resources, patients/family comfortable with the mobility process, and staff understanding of the benefits of early mobilization. We also identified the consistent use of weaning and sedation protocols as key drivers but later deemed these factors out of the scope of this project. The global aim of the project was to improve patient experience and outcomes and to generate cost savings. However, these were also deemed outside the scope of this project.

**Fig. 2.**
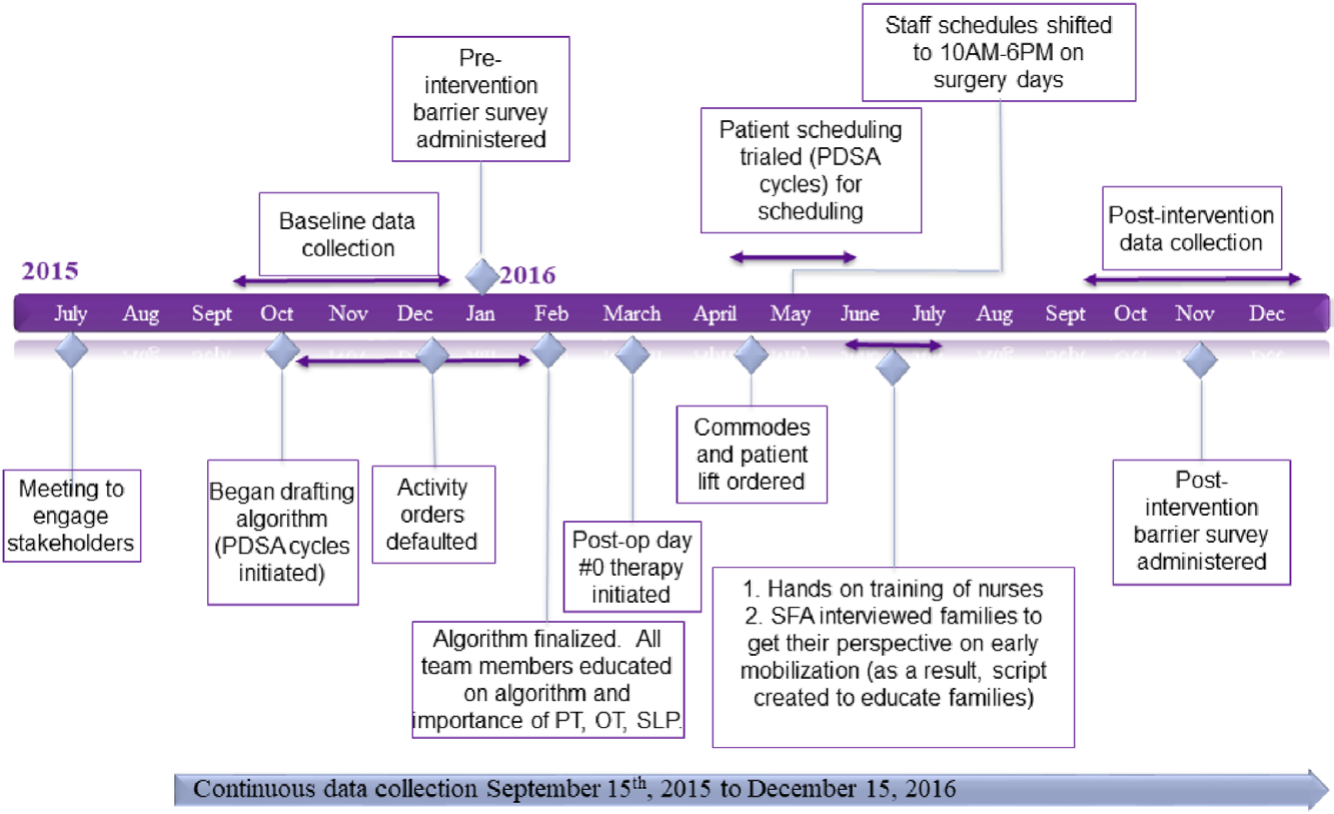
Project timeline.

The primary aim of this project was an 80% mobilization rate of non-MV patients within 12 hours of PICU admission. However, when we found that 67% of patients were admitted to the PICU between 12:00 pm and 8:00 pm, we changed the time frame to within 18 hours of admission to ensure optimal sleep hygiene and availability of rehabilitation for the first mobilization. The timeline for the project is illustrated in Figure 2.

#### Pathway/Algorithm Development

**Fig. 3.**
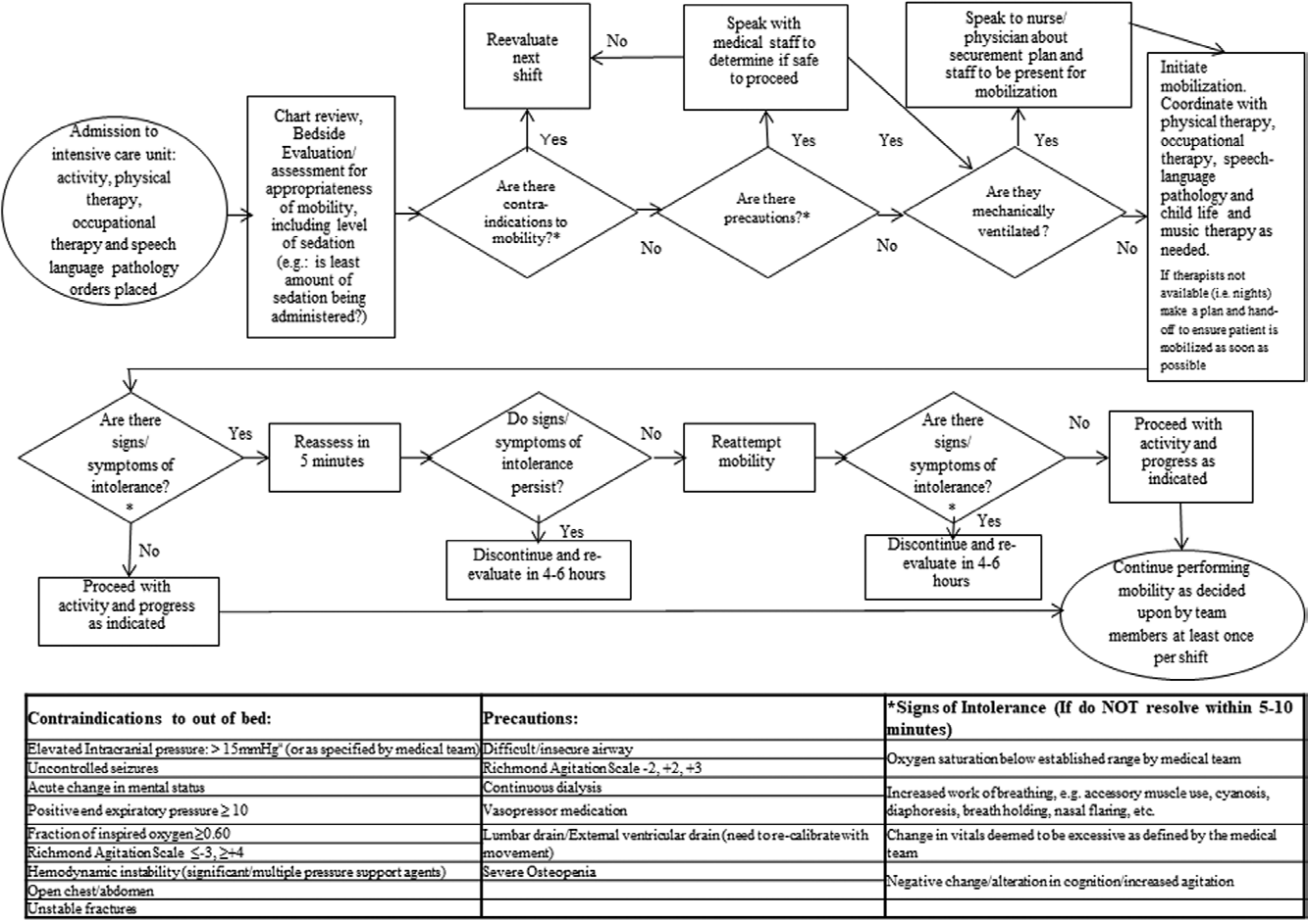
Pediatric intensive care unit algorithm for early mobilization, including contraindications, precautions, and signs of intolerance.

We addressed the accurate identification of patients appropriate for early mobilization as the first key driver. The team obtained consensus on contraindications and precautions^18^ (Fig. 3) and the definition of mobilization. Patients absent contraindications were deemed “ready for mobilization.” We defined “mobilization” as sitting on the edge of the bed, standing, sitting out of bed, and ambulation. Bed mobility was also included only for patients who were on active bed rest but were independently moving around in bed. We defined PICU admission time as the time the patient was admitted to the PICU. If mobilization was contraindicated at that time, the timeline for time to mobilization started upon physician clearance.

To operationalize and guide the decision-making for early mobilization based on those parameters and definitions, we developed an algorithm that included contraindications, precautions, and signs of intolerance (Fig. 2). We assessed the effectiveness of the algorithm by performing Plan-Do-Study-Act^19^ cycles. The team developed the initial algorithm in October 2015, trialed it, and finalized it in February 2016. As part of educating the PICU team about the algorithm, we educated physicians and residents to place orders for patients on postoperative day 0.

#### Accurate and Timely PT, OT, SLP, and Activity Orders

PT, OT, and SLP orders defaulted within the PICU admission order set before the initiation of the project. Activity orders did not default before the project but were defaulted to “up with assistance” as of November 2015.

#### Management of Resources

Concurrent with testing the algorithm, we created and administered a staff survey to identify perceived barriers to mobilization. Fifty-one PICU staff members, including Nurses, PCTs, Physicians, Residents, PTs, OTs, SLPs, and RTs, completed the survey. The survey suggested several barriers, including limited understanding of the benefits of early mobilization, inadequate training, fear of line/tube dislodgment, and lack of resources. To address the barrier of insufficient resources, we increased the commodes from 2 to 7, and patient lifts from 0 to 1. We did not add additional staffing.

To maximize staff efficiency, we trialed scheduling patients for PT, OT, and SLP sessions starting in April 2016. Patients were scheduled for daily PT, OT, and SLP unless contraindicated by the patient’s medical status, or the primary therapist’s recommended intensity. The intensity of services varied from 1 day a week to twice a day; PT and OT treated most patients 4−5 times a week and SLP treated most patients 2 times a week. Ultimately, we determined that due to the complexity of patients and workflow, scheduling was ineffective. Therefore, we abandoned this intervention.

The rehabilitation staff traditionally worked 8:00 am to 4:00 pm. Starting May 2016, 1 therapist started working 10:00 am to 6:00 pm, Tuesday through Thursday, the high-volume surgery days. This change enabled staff to see patients on the day of admission/surgery.

#### Staff Education

In February of 2016, upon finalization of the algorithm, the rehabilitation therapists began attending morning nursing huddles and physician rounds. During rounds, the therapists educated the team about the benefits of early mobilization and ensured that they discussed the patient’s mobilization plan. They also inquired about the possibility of reducing sedation when appropriate so that patients could be mobilized. We initiated the mobilization training for nurses and PCTs in June 2016. Training consisted of PT demonstrating proper mobilization techniques followed by the nursing staff practicing these techniques on the therapist. We identified that training with patients in real-time was more effective, so we shifted to this method in July 2016.

SFAs interviewed patients and families in June to July 2016, focusing discussions on the understanding of the roles of PT, OT, and SLP and the benefits of mobilization. Based on this feedback, we created a script for therapists to ensure they communicated the goals of therapy to patients and their families. The SFAs also shared stories with patients, families, and nurses about how watching the mobilization of their critically ill children in the PICU gave them hope, encouragement, and motivation during difficult times.

### Measures and Analysis

We set the minimum age of 18 months because the physicians felt that by 18 months, most children would be able to follow commands, leading to the safest outcomes. We planned to include younger children if the early mobilization program was found safe and effective. Medical, nursing, and rehabilitation leadership felt that an 80% mobilization rate was reasonable and medically appropriate for this population. We defined the timeframe for early mobilization as 18 hours from admission for non-MV patients and 48 hours for MV patients. Due to limited evidence in this area, we relied on consensus clinical judgment to form our definition. Subsequent research substantiated the need to mobilize critically ill children within 1 to 4 days of admission.^15^

The primary outcome measure was the percentage of patients mobilized early. Process measures were the percentage of patients with PT, OT, SLP, and activity orders and staff perceptions regarding barriers to early mobilization. Secondary outcome measures were PICU and hospital LOS and discharge disposition. We dichotomized the discharge disposition to home (self-care and home health services) and not home (inpatient rehabilitation, skilled nursing, or psychiatric facility). The balancing measure was the number of safety events during mobilization defined as any event that causes harm to the patients, including, but not limited to, dislodgement of lines, unplanned extubations, and falls.

We used a Statistical Process Control chart to track changes in the percentage of patients mobilized over time. Established rules identified for special cause variation, specifically 8 consecutive points above or below the centerline, lead to a midline shift.^20^ Weekly data were not included in the control chart if n <5 that week. To compare the proportional measures (percentage mobilized; received PT, OT, SLP, or activity orders; and discharged home) between the preintervention and postintervention groups, we performed chi-square tests for equality of proportion. To compare the interval measures (hospital and PICU LOS) between groups, we performed nonparametric Independent-Samples Mann–Whitney U tests because both measures had non-normal distributions.

## RESULTS

### Participants

The total number of eligible patients admitted to PICU from September 15, 2015, to December 15, 2016, was 403. The preintervention group consisted of patients admitted to the PICU from September 15, 2015, to December 15, 2015. The postintervention group consisted of patients admitted to the PICU from September 15, 2016, to December 15, 2016. Details about the patients in the pre- and postintervention groups are listed in Table 1. The sample of patients on MV was too small to allow for statistically testing.

**Table 1.**
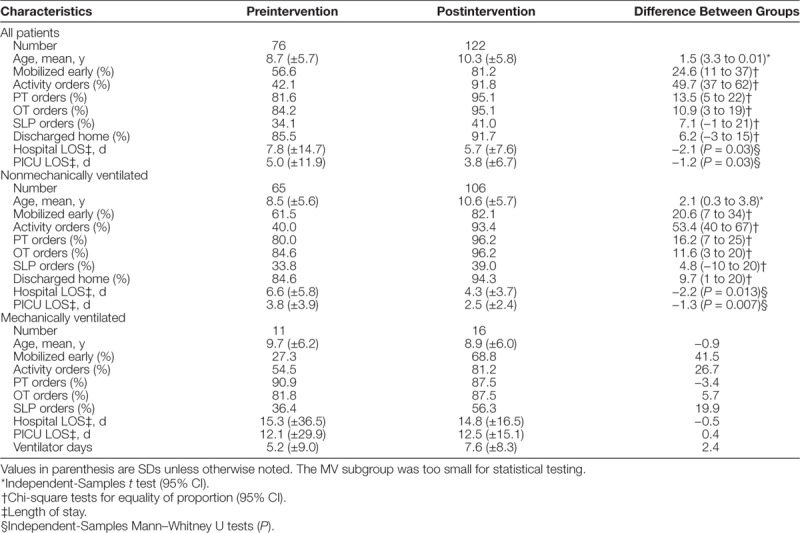
Pre- and Postintervention Group Comparisons

### Safety/Balancing Measure

There were no adverse safety events associated with mobilization.

### Primary Process Measure: Orders

The percentage of patients with activity orders increased 50% (95% CI, 37%–62%), from 42% to 92%; PT orders 14% (95% CI, 5%–22%), from 82% to 95%; OT orders 11% (95% CI, 3%–19%), from 84% to 95%; and SLP orders 7% (95% CI, −1% to 21%), from 34% to 41%. In the non-MV subgroup, percentage of patients with activity orders increased 53% (95% CI, 40%–67%), from 40% to 93%; PT orders 16% (95% CI, 7%–25%), from 80% to 96%; OT orders 12% (95% CI, 3%–20%), from 85% to 96%; and SLP orders 5% (95% CI, −10% to 20%), from 34% to 39%.

### Primary Process Measure: Barriers to Mobilization Survey

Eighty percent (n = 51) of team members completed the preintervention barrier survey (53% nursing staff, 20% rehabilitation therapists, 12% RTs, and 16% physicians/residents; 82% from day shift and 18% from night shift). Seventy percent (n = 43) completed the postintervention survey (58% nursing staff, 28% rehabilitation therapists, 7% RTs, and 7% physicians/residents; 84% from day shift and 16% from night shift). Barriers to early mobilization included lack of resources, appropriate equipment not available, too many lines/drains, patient agitated, confused or delirious, too much coordination needed, patient unsteady at baseline, lack of training/education, patient/family uncomfortable, and other. The most significant concerns identified for mobilizing children pre- and postinterventions were fear of line or drain dislodgement, not trained, unable to manage ventilator/fear of unplanned extubation, and others (Table 2).

**Table 2.**
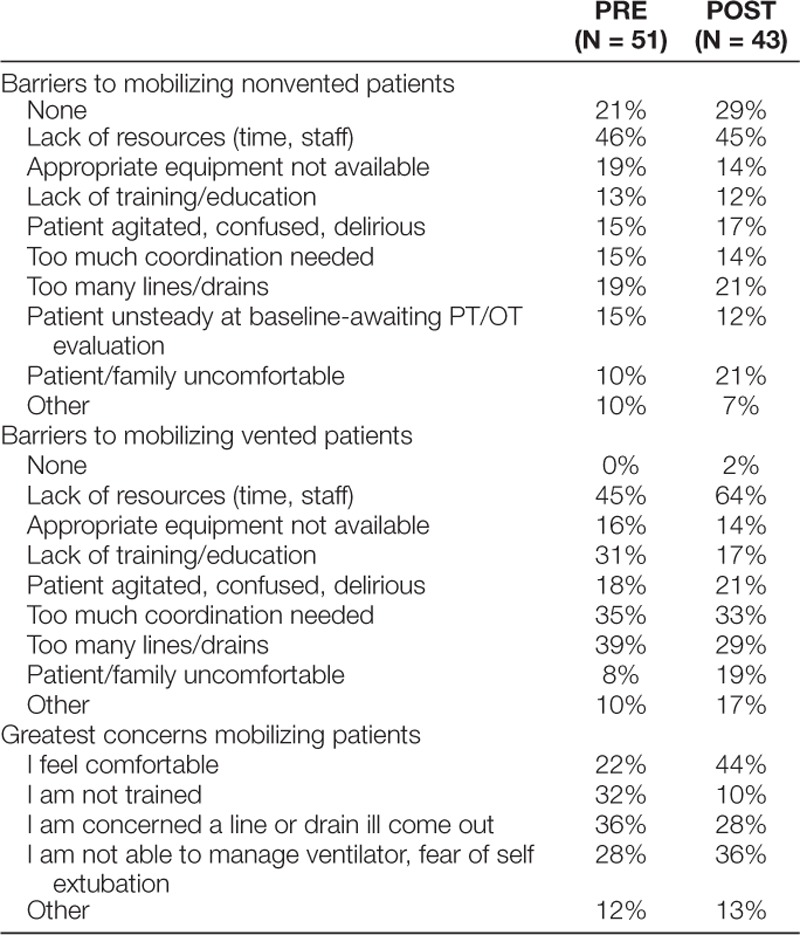
Barriers to Mobilization Survey Results

### Primary Outcome Measure: Mobilization Rate

**Fig. 4.**
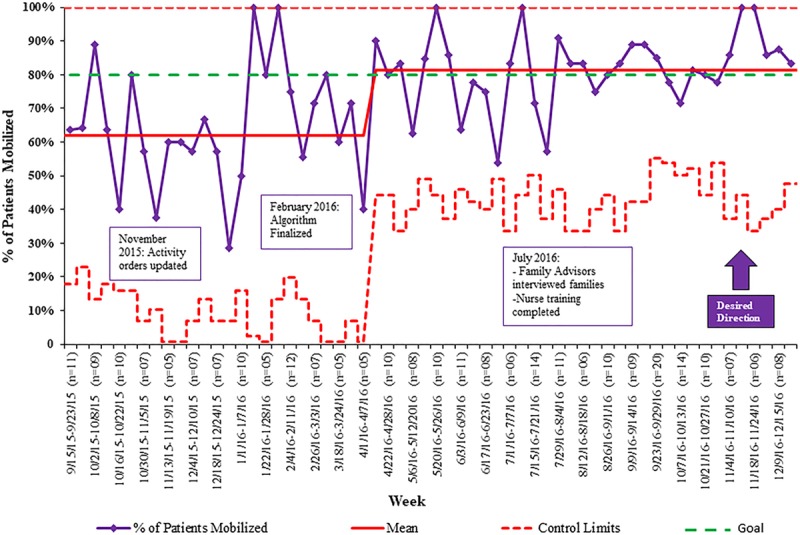
Control chart of primary measure: percent of patients mobilized within the allotted time frame. Allotted time frame defined as 18 hours for mechanically ventilated patients and 48 hours for nonmechanically ventilated patients.

The percentage of patients mobilized early increased 25% (95% CI, 11%–37%) in the postintervention group, 21% (95% CI, 7%–34%) for the non-MV subgroup, and 42% for the MV subgroup. The relative 31% increase of patients mobilized was achieved by April 2016 and sustained for the next 8 months (Fig. 4).

### Secondary Outcome Measure: PICU and Hospital LOS

For non-MV patients, the average hospital LOS decreased from 6.6 (SD = 5.8) to 4.3 (SD = 3.7), and the average PICU LOS decreased from 3.8 (3.9) to 2.4 (2.4) days (Table 1). Both changes were statistically significant (<0.05). For MV patients, the average hospital LOS decreased from 15.3 (SD = 36.5) to 14.8 (SD = 16.5), and PICU LOS increased from 12.1 (SD = 29.9) to 12.5 (15.1). The sample size of MV patients was too small for statistical testing.

### Secondary Outcome Measure: Discharge Disposition

The percentage of patients who went home (versus another facility) increased 6% (95% CI, -3% to 15%) for the combined MV/non-MV postintervention cohort and increased 10% (95% CI, 1%–29%) for the non-MV patients. The percentage of patients who went home (versus another facility) increased 7% (95% CI, -2% to 17%) for the combined MV/non-MV early mobilization cohort and 9% (95% CI, −1% to 19%) for the non-MV patients.

## DISCUSSION

The results of this quality improvement project suggest that early mobilization of patients in the PICU is feasible, safe, and potentially effective. We exceeded our goal of mobilizing 80% of patients within the established time frame and observed significant improvements in our process and outcome measures.

The comparison of the pre- and postintervention surveys suggested that some perceptions of barriers to early mobilization changed. Based on the postintervention surveys, nurses and therapists had no perceived barriers to mobilization, and staff felt more comfortable mobilizing critically ill patients. They perceived patient and family comfort level with early mobilization was more of a barrier in the postintervention phase. This perception may be related to the greater level of engagement of patients and families in the mobilization process as a result of this initiative. We will revise our education to ensure that patients and families are comfortable with the mobilization process. Staff had more significant concerns regarding unplanned extubation in the postintervention phase. As with patient and family discomfort, this concern may also be associated with the increased frequency of mobilization of this population. We have addressed this concern in on-going education sessions.

The percentage of patients with activity, PT, OT, and SLP orders increased following the interventions. The increase of patients with activity, PT, and OT orders at admission may have contributed to the improved early mobilization rate because it decreased the time therapists had to spend clarifying the patient’s mobility status. The increased orders could not explain all of the improvement, for the following reasons: (1) When inspecting the statistical process control chart, the mobilization rate did not seem to change meaningfully after the activity orders defaulted; and (2) the percentage PT and OT orders were high at the outset. Despite being defaulted at program onset, the reason these orders increased was that physicians sometimes manually deselecting these elements of the order set before the start of the program. Although the increase in the percentage of SLP orders did not reach statistical significance, we believe the role of SLP was vital because they assisted patients in communicating their needs, goals, fears, and sense of accomplishment. Educating the nurses and PCTs to mobilize patients also appeared helpful.

The role of the SFAs was invaluable. The interventions they lead provided a unique insight to the team and help empower patients, families, and caregivers to advocate for their loved ones. The feedback that the advisors received from the families also allowed the mobilization team to interact with patients and their caregivers in a more effective manner.

This study has some limitations. Due to the manual data collection process, and variable staff on the weekends, data collection was not as consistent, most specifically, on the weekends. If the time of the first mobilization was unknown, the patients were excluded, which may have influenced our results in either direction. Also, because of the small number of patients on ventilators, we were unable to make a meaningful comparison between the pre- and postintervention groups for MV patients.

We deemed the ability to optimize sedation and weaning protocols outside the scope of this project. We will assess this issue more closely in future projects.

## CONCLUSIONS

Most early mobility studies focus on the adult population. Results of this project demonstrate that when applying improvement science methodology, early mobilization of critically ill children in a PICU can be safe, feasible, and effective. It may be correlated with decreased LOS, and an increased percentage of children discharged home. Larger, multisite studies of dissemination and implementation would be helpful in this high-risk population.

## DISCLOSURE

The authors have no financial interest to declare in relation to the content of this article.

## ACKNOWLEDGMENTS

Tami Altschuler, MS, CCC-SLP, Rashad Bizzell, BS, Ashley Carr, MS, OTR/L, Jennifer Daly, Aaron Janson, MD, Naomi Linder-Perlman, JD, Lucy Pereira-Argenziano, MD, Stacey Schneider, MA, ATR, CCLS, LCAT, Lauren Selikoff, RN, BSN, Lauren Simon, PT, DPT, NCS, Tina Tan, MS, CCC-SLP, BCS-S, The Sala Institute for Child and Family-Centered Care, and Hassenfeld Children’s Hospital at NYU Langone Health involved in assistance with the study.
